# Authors’ Reply: Modernizing Gender, Sex, and Sexual Orientation Data Through Engagement and Education

**DOI:** 10.2196/52286

**Published:** 2023-11-15

**Authors:** Roz Queen, Karen L Courtney, Francis Lau, Kelly Davison, Aaron Devor, Marcy G Antonio

**Affiliations:** 1 School of Health Information Science University of Victoria Victoria, BC Canada; 2 Chair in Transgender Studies University of Victoria Victoria, BC Canada; 3 School of Information University of Michigan Ann Arbor, MI United States

**Keywords:** data sharing, digital health systems, digital health, gender, sex, and sexual orientation, electronic health records, GSSO, health information standards, LGBT health, LGBT, policy

The authors [1] agree with the letter to the editor [2] that education and engagement are necessary for the successful modernization of gender, sex, and sexual orientation in digital health systems. The participants in our knowledge translation project tended to focus on the need for more short-term education initiatives with providers, staff, patients, and family members related to implementing changes. We appreciate that the authors of the letter identify the need for long-term education initiatives such as changes to health care provider curriculum as well as health education provided to the public. We fully and strongly encourage the development, implementation, and sharing of education initiatives as an important part of modernization efforts. SOGI Nursing is a great example of an educational initiative [3].
